# CRISPR/Cas9-mediated gene editing of *vacuolar ATPase subunit d* mediates phytohormone biosynthesis and virus resistance in rice

**DOI:** 10.3389/fpls.2023.1122978

**Published:** 2023-02-01

**Authors:** Qinghua Lu, Xiangwen Luo, Xiao Yang, Tong Zhou, Yu Zhang, Ying Lan, Deyong Zhang, Limin Zheng, Yixin Li, Li Li, Songbai Zhang, Yong Liu

**Affiliations:** ^1^ Longping Branch of Biology College, Hunan University, Changsha, China; ^2^ State Key Laboratory of Hybird Rice, Hunan Academy of Agricultural Sciences, Changsha, China; ^3^ Key Laboratory of Food Quality and Safety, Institute of Plant Protection, Jiangsu Academy of Agricultural Sciences, Nanjing, China

**Keywords:** CRISP/Cas9, vacuolar ATPase subunit d, phytohormone biosynthesis, virus resistance, rice

## Abstract

Vacuolar ATPases (V-ATPases) are proton pumps for proton translocation across membranes that utilize energy derived from ATP hydrolysis; OsV-ATPase subunit d (OsV-ATPase d) is part of an integral, membrane-embedded V0 complex in the V-ATPase complex. Whether OsV-ATPase d is involved in phytohormone biosynthesis and resistance in rice remains unknown. The knockout mutants of *OsV-ATPase d* in rice were generated using the CRISPR/Cas9 system, and mutation of *OsV-ATPase d* did not show any detrimental effect on plant growth or yield productivity. Transcriptomic results showed that *OsV-ATPase d* is probably involved in mediating the biosynthesis of plant hormones and resistance in rice. Compared to wild type, mutation of *OsV-ATPase d* significantly increased JA and ABA biosynthesis and resistance against *Southern rice black-streaked dwarf virus* (SRBSDV), but it decreased resistance against *Rice stripe virus* (RSV) in rice. The data presented in this study reveal that *OsV-ATPase d* mediates phytohormone biosynthesis and virus resistance in rice and can be selected as a potential target for resistance breeding in rice.

## Introduction

Rice (*Oryza sativa*) is one of the most important staple foods for more than half of the world’s population. However, many viral diseases have severely challenged rice production and have increased the risk to food security in many global areas (Jones and Dangl, 2006; Mandadi and Scholthof, 2013; Nicaise, 2014; Baulcombe, 2019; ). For example, *Southern rice black-streaked dwarf virus* (SRBSDV) is severely epidemic and has caused 30-50% rice yield losses in southern China and Southeast Asia in the last decade (Hoang et al., 2011; Cheng et al., 2013; Zhang et al., 2013; Zhou et al., 2013; ). *Rice stripe virus* (RSV) is one of the most destructive virus affecting rice production, and it is wide-spread throughout East Asia, especially in China, Japan and Korea (Hu et al., 2020). Although SRBSDV and RSV has been successfully controlled by international cooperation, it still exists in the majority of rice-producing areas of eastern China, with periodic outbreaks in a few rice-producing areas and the potential for additional widespread outbreaks (Lv et al., 2017; Alonso et al., 2019). One of the most effective strategies to prevent viral diseases is growing resistant or tolerant varieties; nevertheless, almost all cultivated rice varieties are susceptible to SRBSDV (Wang et al., 2017; Yu et al., 2017; Zhou et al., 2021).

CRISPR-Cas9 based genome editing is increasing concern as a promising technique to induce resistance against DNA and RNA viruses in crop plants, and also can be explored for a variety of agronomic traits in agriculture crops (Hinge et al., 2021). And CRISPR/Cas-based genome editing is an alternative method for accelerating rice improvement (Ma et al., 2015). The availability of rice reference genome sequences and the CRISPR/Cas9-editing system has made it possible to develop disease-resistant or disease-tolerant rice by precisely editing endogenous genes (Huang et al., 2012; Yang et al., 2013; Jackson, 2016; Wing et al., 2018; Zhao et al., 2020).

Vacuolar ATPases (V-ATPases) are proton pumps for proton translocation across membranes that utilize energy derived from ATP hydrolysis (Forgac, 2007; Mazhab-Jafari and Rubinstein, 2016). In eukaryotic cells, V-ATPases are multisubunit complexes that mediate the pH of many intracellular organelles, including vacuoles and endosomes (Forgac, 2007). V-ATPase has been reported to regulate defense against viral infection in mammals, and V-ATPase activity is critical in mammal-virus interactions (Hunt et al., 2011; Jang et al., 2018). The V-ATPases of endosomes play pivotal roles in the successful entry and release of the viral genome into the cytoplasm for most human viruses, including *Human coronavirus NL63* (HCoV-NL63), influenza viruses, *Zika virus* (ZIKV), *Dengue virus* (DENV), and *Sindbis virus* (SINV) (Hunt et al., 2011; Jang et al., 2018; Kao et al., 2018; Milewska et al., 2018). Nevertheless, the subunits of V-ATPase present in the vacuoles during plant−virus interactions have never been studied. The latest study demonstrated that V-ATPase also regulates plant−virus interactions. For the first time, BSMV replicase was reported to competitively bind to subunit B2 of V-ATPase to detach subunit B2 from the tonoplast into the cytosol, leading to impairment of the connection between the V1 and V0 complexes. This severed connection disrupted the formation of V-ATPase on the tonoplast, which inhibited the activity of V-ATPase, increased the vacuolar pH in plants, and promoted infection by BSMV and multiple viruses, including *Lychnis ringspot viru*s (LRSV), *Cucumber mosaic virus* (CMV), or *Potato virus X* (PVX) (Yang et al., 2021). However, the other subunits of V-ATPase involved in plant−virus interactions have never been studied.

The pathogens *Sarocladium oryzae* and *Pseudomonas fuscovaginae* cause rice sheath rot and produce cyclic lipopeptides to inhibit membrane-bound H^+^-ATPase pumps in the rice plant, resulting in reduced abscisic acid (ABA), jasmonate acid (JA) and auxin levels and grain yield in rice (Peeters et al., 2020). This suggests that H^+^-ATPase is probably involved in regulating the plant hormone pathway. Plant hormones are pivotal for biotic and abiotic resistance, and rice hormones have diverse functions in rice resistance against different viruses (Yan and Xie, 2015; Xie et al., 2018; Zhang et al., 2020). Therefore, *OsV-ATPase d* might be an alternative target for gene editing by CRISP/Cas9 to enhance viral resistance in rice.

In this study, the function of *OsV-ATPase d*, which is a rice gene encoding the protein V-ATPase d that functions as subunit d of the membrane-embedded V0 complex of V-ATPase in rice plants, was characterized by using knockout rice lines. The results showed that knocking out *OsV-ATPase d* in rice had no detrimental impact and differentially mediated resistance against the plant RNA viruses RBSDV and RSV and phytohormone biosynthesis in rice. To the best of our knowledge, this is the first report of a protein with a classical function as a proton pump for proton translocation during the regulation of vacuolar acidification being involved in different methods of plant defense against plant RNA viruses and the regulation of phytohormone biosynthesis.

## Materials and methods

### Plant growth and virus inoculation

Rice cultivar *Oryza sativa* L. *japonica*. Nipponbare (NIP) was used in this study. The cultivar Nipponbare is highly susceptible to SRBSDV and RSV (Xu et al., 2014; Yang et al., 2018b). NIP was used to produce transgenic rice. SRBSDV-infected plants were kindly provided by Professor Guohui Zhou and Tong Zhang (South China Agricultural University Guangzhou, China). Rice seedlings were grown in a greenhouse at 26 to 28°C with a 14-h light/10-h dark cycle under artificial light. Rice plants infected with SRBSDV and RSV were cultivated in an experimental field in Changsha and Nanjing, respectively, under natural long-day conditions. Viruliferous or virus-free planthoppers were reared on healthy rice seedlings (Wuyujing No. 3) in glass beakers at 25°C.

SRBSDV was transmitted by the white backed planthopper (*Sogatella furcifera*) at approximately the 1.5-leaf-stage of rice seedling. To obtain viruliferous insects, nymphs were reared on virus-infected rice plants for 2 days, and viruliferous or virus-free nymphs were transferred to each experimental rice plant to feed for 3 days, after which the planthoppers were removed. The proportions of healthy plants were calculated 30 days after inoculation. The percentage of about 30 plants infected by virus (viral incidence) of each of triplication was determined following specific quantitative RT−PCR of samples of each plant using virus-specific primers (**Table S1**) and western blotting using SRBSDV P8 polyclonal antibody.

RSV was transmitted experimentally to rice plants by the small brown planthopper (*Laodelphax striatellus*) at approximately the 1.5-leaf-stage of rice seedling. To obtain viruliferous insects, nymphs were reared on virus-infected rice plants for 2 days, and viruliferous or virus-free nymphs were transferred to each experimental rice plant to feed for 3 days, after which the planthoppers were removed. The proportions of healthy plants were calculated 30 days after inoculation. The percentage of about 30 plants infected by virus (viral incidence) of each of triplication was determined following specific quantitative RT−PCR of samples of each plant using virus-specific primers (**Table S1**).

### Vector construction and agrobacterium-mediated transformation in rice

The binary CRISPR/Cas9 vector pYLCRISPR/Cas9-MH and corresponding gRNA vector pYLgRNA-U3 were provided by Yaguang Liu at South China Agricultural University. Specific single guide RNAs (sgRNAs) targeted to *OsV-ATPase d* were selected and constructed and used to transform the rice cultivar NIP by Agrobacterium-mediated transformation as previously described (Ma et al., 2015). The transgenic rice lines were selected based on hygromycin resistance. The primers used for vector construction are listed in **Supplementary Table S1**.

### Genomic DNA extraction and mutation detection

The genomic DNA was extracted from young leaves of T0-T2 transgenic plants by CTAB reagent, which was then used to amplify specific fragments in the *OsV-ATPase d* gene using primers flanking two targeted sites (**Table S1**). PCR was conducted under the following conditions: 94°C for 5 min; 94°C for 30 s, 56°C for 30 s, and 72°C for 1 min (35 cycles); and 72°C for 10 min as the final extension. PCR products were directly sequenced using the Sanger method by Sangon Biotech Company (Shanghai, China). The transgenic plants were also verified as Cas9-free with primers specific for Cas9 (**Table S1**) (Ma et al., 2015).

### Measurement of agronomic traits of rice

Seeds from fully mature rice were collected and dried. Seeds from each rice plant were randomly selected. Seed weight was measured using an electronic balance. Plant height and spike length were measured with a steel ruler.

### RNA library construction, sequencing, and analysis

Total RNA was isolated from leaves of 15-day-old rice with a plant RNA purification reagent kit (Invitrogen, USA). The concentration, quality, and purity of the RNA were detected with an Agilent 2100 Bioanalyzer RNA 6000 Nano kit (Agilent, USA). RNA libraries were constructed with a TruSeq™ RNA Sample Prep kit (Illumina, USA) and sequenced by OE Biotech Company (Shanghai, China) on an Illumina HiSeq 4000. The clean reads were mapped to the reference genome using HISAT2 (Kim et al., 2015), and DEGs were identified using DESeq (Frazee et al., 2014). GO enrichment and KEGG (Kanehisa et al., 2008) pathway enrichment analyses of DEGs were performed using R based on the hypergeometric distribution.

### Total RNA isolation and quantitative reverse transcription polymerase chain reaction (qRT−PCR)

Total RNA was isolated from rice leaf samples (100 mg tissue per sample) using TRIzol reagent (TIANGEN Biotech, Beijing, China). The concentration of total RNA in each sample was determined using a NanoDrop 2000 Spectrophotometer (Thermo Fisher Scientific, Wilmington, DE, USA). cDNA was synthesized using 1 µg total RNA per 20 µL reaction using the PrimeScript™ RT Reagent Kit with gDNA Eraser (Takara, Dalian, China). Quantitative RT-PCRs were performed on an ABI PRISM 7500 device using a SYBR Premix ExTaq RT−PCR Kit (Takara). Relative transcript levels were calculated by the 2^-ΔΔ^CT method as previously described (Rao et al., 2013), and the rice ubiquitin gene (Os03g0234350) was used as an internal control. The primers for quantitative RT−PCR analysis are listed in **Table S1**.

### Immunoblot assay of SRBSDV P8 protein

Assays of SRBSDV P8 proteins were performed as described previously (Yang et al., 2018b). Total protein was isolated from leaf tissues at 30 dpi and separated in 15% SDS−PAGE gels. The protein bands were blotted onto polyvinylidene difluoride (PVDF) membranes followed by protein detection using an SRBSDV P8 polyclonal antibody. Protein loading was estimated through Coomassie Brilliant Blue staining.

### Quantification of endogenous phytohormones in tissue samples

The assay was determined following a previously published method (Novák and Floková, 2018) Rice leaf samples (50 mg each) were collected from the assayed plants at 30 dpi, ground individually in liquid nitrogen, and then homogenized in 1 ml extraction buffer containing isopropanol/H_2_O/hydrochloric acid (200:100:0.2). The crude leaf extracts were incubated at -20°C for 12 h and then ultrasonicated for 30 min in an ice bath, followed by the addition of 1 mL of dichloromethane and 1 μL of 300 ng/mL double internal standard samples (succinic acid - 2,2,3,3 - d4 and Lyso PC17:0). The organic phase was evaporated to dryness in vacuo, dissolved in 200 μl methanol/H_2_O (5:95, including 10 ng/ml 2-cl-phe), ultrasonicated for 3 min in an ice bath, and then centrifuged at 13 000 rpm for 10 min at 4°C. The supernatant of each sample was filtered with a 0.22 µm organic filter membrane and then tested by OE Biotech Company (Shanghai, China) using HPLC−MS (AB Exion coupled with AB Sciex Qtrap 6500+) with an ACQUITY UPLC HSS T3 chromatographic column (100 mm×2.1 mm, 1.8 μm).

### Statistical analysis

All the experiments conducted in this study were performed in triplicate to quintuple. The results of the experiments are presented as the means of three to five independent experiments ± their standard deviations (SDs). Statistical analyses were performed using DPS 19.05 software (Tang and Zhang, 2013) and Student’s *t* tests (Krzywinski and Altman, 2013).

## Results

### CRISPR/Cas9-engineered mutations in Osv-ATPase d had no effect on rice agronomic traits

**Figure 1:**
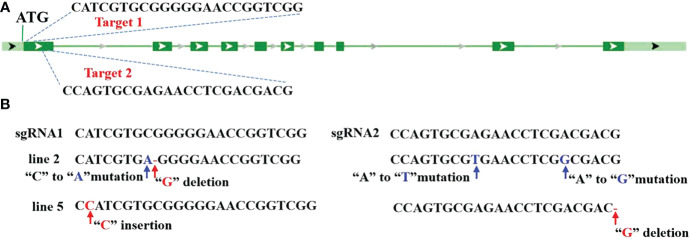
CRISPR/Cas9-mediated editing of *Osv-ATPase d* **(A)** Illustration of *Osv-ATPase d* gene structure and the editing target. **(B)** Mutant sites at *Osv-ATPase d.*.

In this study, CRISPR/Cas9-based genome-editing technology was employed to edit *OsV-ATPase d* in Nipponbare (*Oryza sativa* L. cv. japonica, NIP), which is highly susceptible to SRBSDV and *rice stripe virus* (RSV) (Zhang et al., 2019). Two guide RNAs were designed to target the first exon of *OsV-ATPase d* by CRISPR Design (http://cripsr.mit.edu) (**Figure 1A**). Specific single guide RNAs (sgRNAs) targeted to *OsV-ATPase d* were selected and constructed by universal primers (**Table S1**) and used to transform the rice cultivar NIP by *Agrobacterium*-mediated transformation. Five independent T0 lines were found to carry heterozygous mutations in *OsV-ATPase d*. From the T1 segregation population, two CAS9-free homozygous mutants with knock-out of *OsV-ATPase d* (hereafter named line 2 and line 5) were identified. Conventional Sanger sequencing verified that a “G” deletion resulted in a frameshift mutant and three nucleotide site mutations in line 2, and a “C” insertion resulted in a frameshift mutant with a “G” deletion in line 5 (**Figure 1B**).

**Figure 2:**
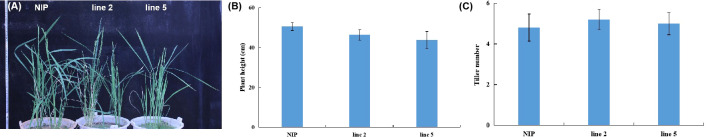
Morphology of plants of the knock-out mutants of *Osv-ATPase d* and the wild type. **(A)** Plants at 60 days of age. **(B)** Plant height; **(C)** Tiller number.

The growth trial of editing line 2 and line 5 grown in pots under greenhouse conditions showed normal growth with no morphological differences when compared to wild-type plants at 60 days of age (**Figures 2A-C**). No adverse effect was observed regarding the yield characteristics spike length, number of spikelets, grain number per spike and 1000-grain weight between edited mutants and wild-type plants (**Table 1**). These results suggested that there was no detrimental impact of knocking out *OsV-ATPase d* in rice.

**Table 1:** Agronomic traits of the knock-out mutants of *OsV-ATPase d* and the wild type.

Sample	Spike length (cm)	Number of spikelets	grain number per spike	1000-grain weight (g)
line 2	20.86 ± 0.52a	10.60 ± 0.81a	116.80 ± 5.22a	19.66 ± 1.45a
line 5	20.26 ± 0.56a	9.67 ± 0.67a	114.33 ± 1.45a	19.76 ± 1.18a
NIP	19.37 ± 0.47a	9.67 ± 0.33a	104.00 ± 4.04a	20.91 ± 0.91a

Letters indicate significantly different values using the Student’s t test (ρ < 0.05).

### The transcriptome profile of more genes was upregulated in *OsV-ATPase d* knockout rice

**Figure 3:**
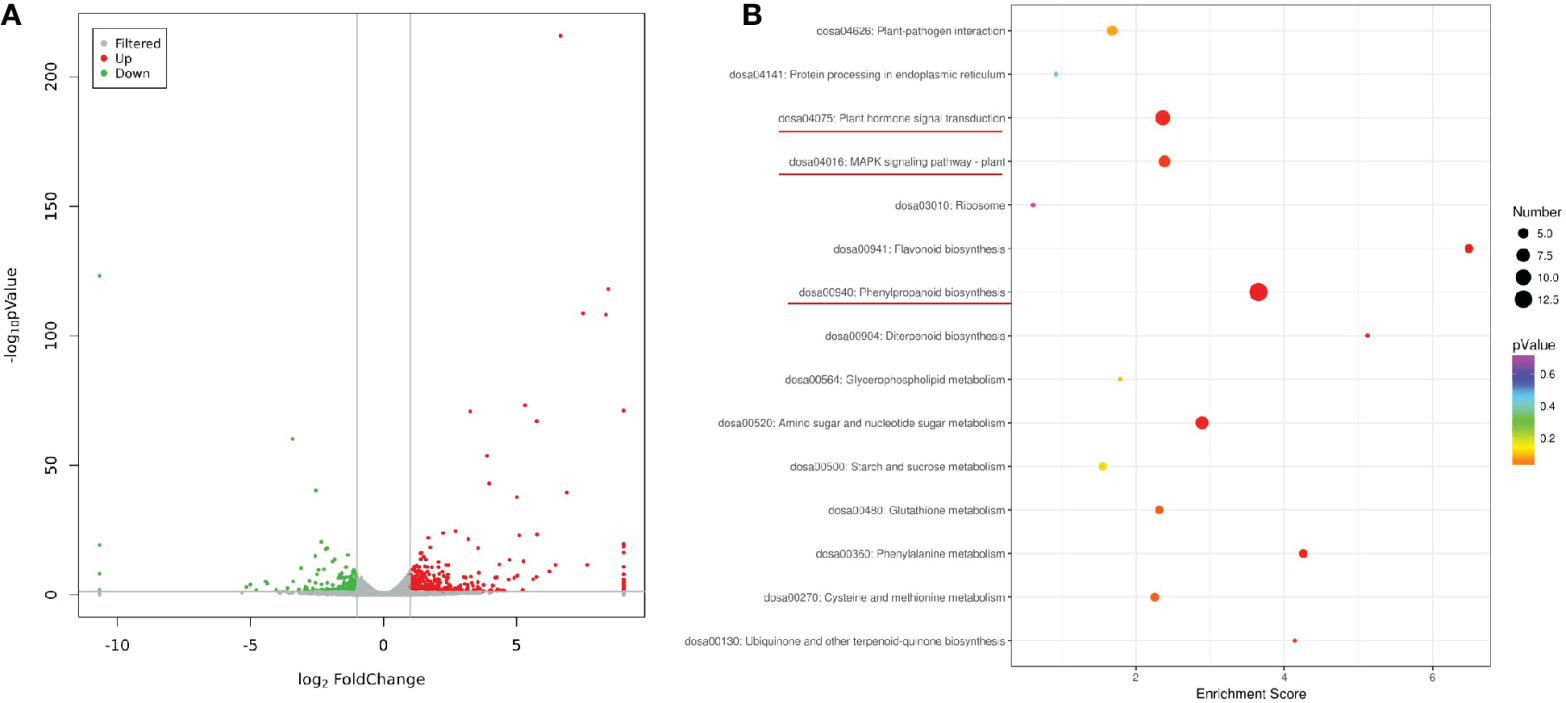
DEGs analysis and KEGG enrichment analysis of the DEGs. **(A)** The volcano map presents the differentially expressed genes (FDR < 0.05 and >= 2-fold change) between the line 5 and the wild type based on leaf transcriptome analysis. **(B)** KEGG pathways with enrichment of significantly upregulated and downregulated genes. Plant hormone biosynthesis genes and plant defense genes are underlined in red.

To gain insight into the functional profiles of *OsV-ATPase d* in rice, the transcriptomic response (dataset was permanently deposited in GenBank with accession number: PRJNA753714) of editing line 5 plants was comparatively analyzed with that of wild-type plants. The transcriptomic sequencing yielded approximately 277.25 M total clean reads, with a mapping ratio of 91.73% - 92.48% after quality control. The average number of clean reads obtained from editing line 5 and NIP samples was approximately 46.86 M and 45.56 M, respectively (**Table S2**). A global analysis of mapped reads uncovered 35,772 expressed genes in the rice leaves. Compared with wild-type plants, a total of 664 differentially expressed genes (DEGs) were induced in the editing line 5 seedlings (15 days old) using the criteria of log2 FC >1 and <−1 under an adjusted *ρ* < 0.05, and among these DEGs, 443 were upregulated and 221 were downregulated in editing line 5 (**Figure 3A**). Gene Ontology (GO) analysis of the upregulated DEGs showed enrichment of 3 biological pathways associated with resistance, including the jasmonic acid-mediated signaling pathway, defense response, and ethylene-activated signaling pathway (**Figure S1**). Nevertheless, GO enrichment pathways of the downregulated DEGs were not related to the defense response but included the regulation of stomatal closure, extracellular region and O-acyltransferase activity (**Figure S2**). Similarly, KEGG annotation of DEGs revealed the enrichment of pathways related to resistance, including phenylpropanoid biosynthesis, plant hormone signal transduction and the MAPK signaling pathway (**Figure 3B**). These findings showed that *OsV-ATPase d* is probably involved in mediating the biosynthesis of plant hormones and resistance to pathogens of rice and may be involved in the molecular mechanisms of both pathways.

### qRT−PCR validation of RNA-seq expression changes

**Figure 4:**
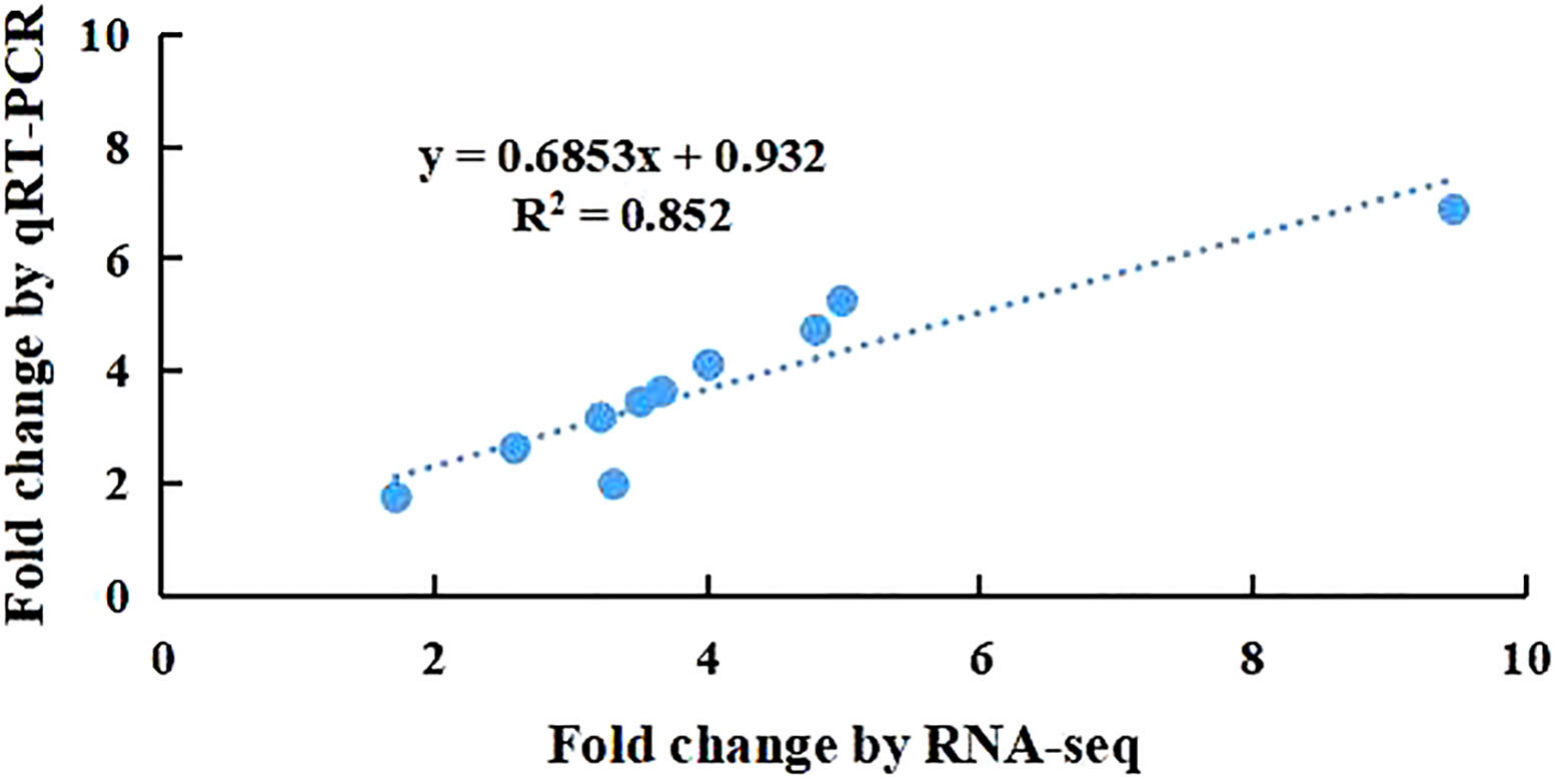
Comparison of the Fold Changes of 15 selected transcripts using RNA-Seq and qRT-PCR. Each blue point represents a chosen gene used in the validation of the RNA-Seq results.

To validate the repeatability of the expression fold changes obtained in the RNASeq dataset, 10 selected genes (*OsLBD12*, *OsATL79*, *OsGSTT3*, *OsMYB5*, *OsAOX1B*, *OsRGA5*, *OsNCED4*, *OsCHX15*, *OsCKX1*, and *OsWRKY27*) were verified by qRT−PCR. As shown in **Figure 4**, the high correlation coefficient (*R^2^* = 0.85) of the expression fold changes of selected genes from qRT−PCR was compared with those obtained from RNA-Seq, which confirmed that the RNA-Seq data are reproducible.

### Knockout of *OsV-ATPase d* increased endogenous ABA and JA biosynthesis in rice

Transcriptomic analysis showed that *OsV-ATPase d* is involved in plant hormone mediation; thus, the plant hormones in editing line 5 and the wild type were then quantified by ultrahigh-performance liquid chromatography-triple quadrupole mass spectrometry (UPLC−MS/MS). As expected, *OsV-ATPase d* was indeed involved in mediating plant hormone biosynthesis. Compared with wild-type plants, editing line 5 showed significantly increased JA and ABA biosynthesis, but there was no effect on the biosynthesis of five other plant hormones, including 1-aminocyclopropanecarboxylic acid (ACC), indoleacetic acid (IAA), salicylic acid (SA) and *trans*-zeatin (tZ) (**Table 2**).

**Table 2:** Quantitative determination of endogenous plant hormones by a UPLC-MS/MS system.

Sample	ABA (ng/g)	ACC (ng/g)	IAA (ng/g)	JA (ng/g)	SA (ng/g)	tZ (ng/g)
line 5	18.37 ± 0.95a	222.56 ± 9.67a	12.39 ± 0.12a	204.05 ± 5.74a	3157.16 ± 121.79a	18.15 ± 2.09a
NIP	10.71 ± 0.62b	213.41 ± 75.42a	9.58 ± 1.43a	124.23 ± 14.73b	3672.21 ± 940.16a	17.47 ± 4.17a

Letters indicate significantly different values using the Student’s t test (ρ < 0.05).

### Genes involved in ABA and JA biosynthesis and signal transduction were upregulated in Osv-ATPase d knockout rice

**Figure 5:**
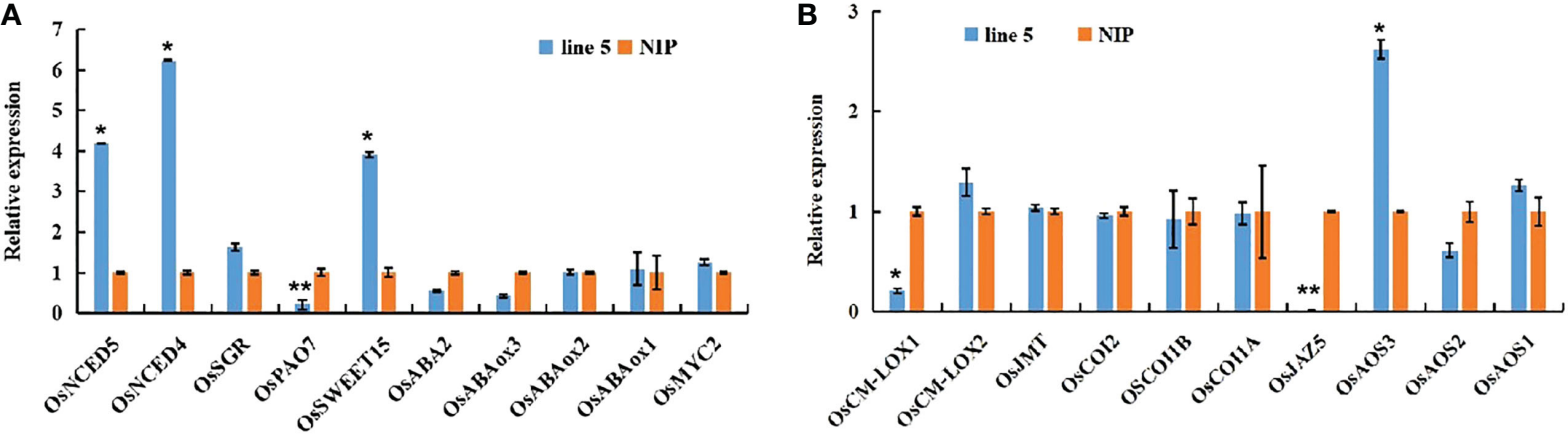
Expression of ABA- and JA-related genes at the seedling stage. **(A)** expression of ABA-related genes including biosynthetic genes *OsNCED4* (LOC_Os07g0154201), *OsNCED5* (LOC_Os12g0617250), *OsPAO7* (LOC_Os09g0368500) and *OsABA2* (LOC_Os04g0452500) and inactivation genes *OsABAox1* (LOC_Os02g0703600), *OsABAox2* (LOC_Os08g0472800) and *OsABAox3* (LOC_Os09g0457250), and signaling transduction genes including *OsSGR* (LOC_Os09g0532000), *OsSWEET15* (LOC_Os02g0513100), and positive regulator *OsMYC2* (LOC_Os10g0575000). **(B)** expression of JA-related genes including biosynthetic genes OsAOS1 (LOC_Os03g0767000), OsAOS2 (LOC_Os03g0225900), OsAOS3 (LOC_Os02g0218700) and OsJMT (Os06g0313440) and signaling transduction genes including *OsCOI1A* (LOC_Os01g0853400), *OsCOI1B* (LOC_Os05g0449500), *OsCOI2* (LOC_Os03g0265500), *OsJAZ5* (LOC_ Os07g0153000), *OsCM-LOX1* (LOC_Os12g0559934) and *OsCM-LOX2* (LOC_Os02g0194700). Error bars indicate means ± SD (n = 3). *ρ ≤ 0.05, **ρ ≤ 0.01 by Student’s *t* test.

**Figure 6:**
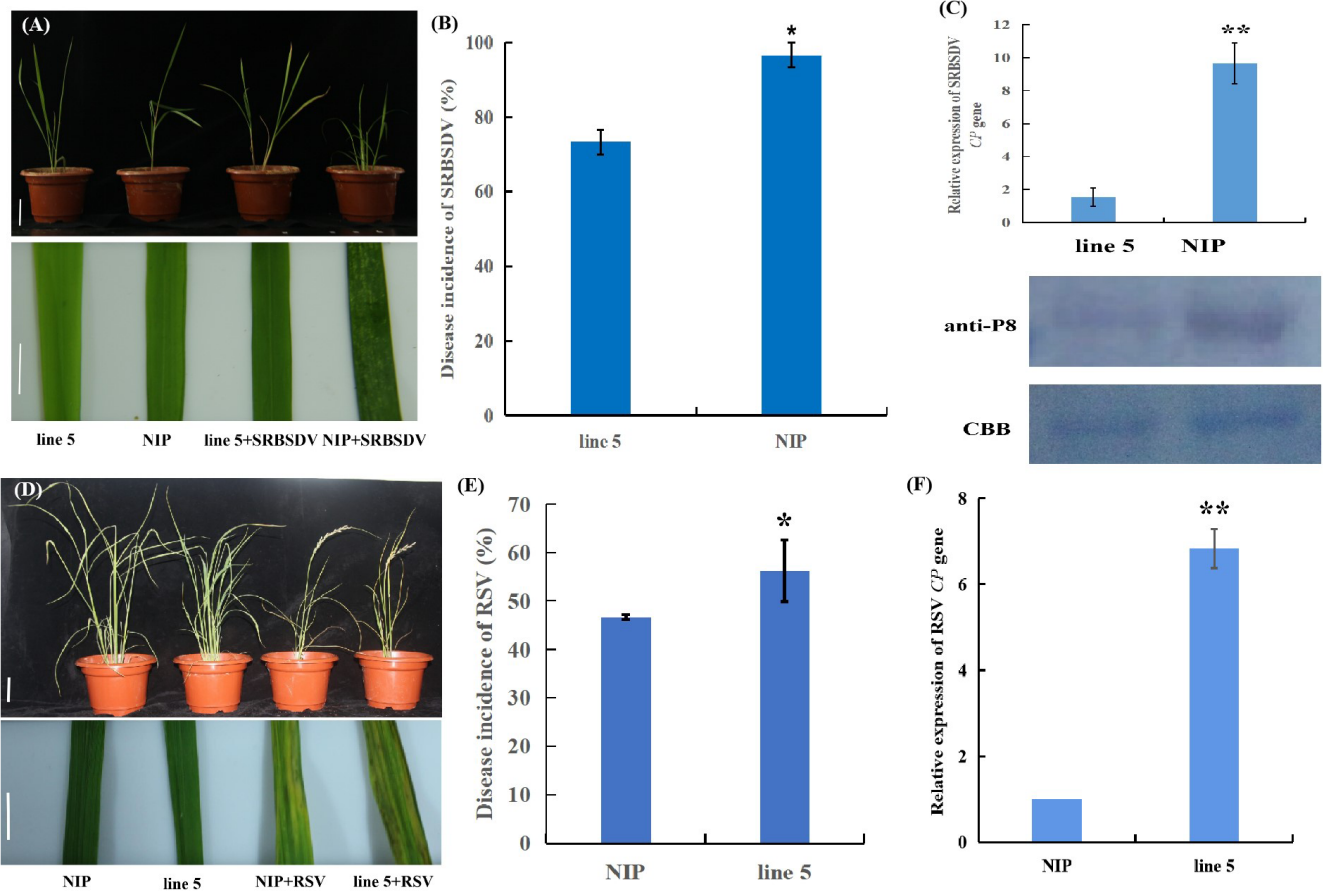
*Osv-ATPase d* differently modulates rice resistances to SRBSDV and RSV. **(A)** Representative images of mock-inoculated or *southern rice black-streaked dwarf virus* (SRBSDV)-infected NIP and line 5 plants. Bar = 2 cm (top). Bar = 1 cm (bottom). **(B)** The percentages of SRBSDV-infected NIP and line 5 plants. **(C)** Detection of SRBSDV levels by quantitative RT-PCR of *CP* gene RNA expression levels and by western blotting using antibodies against the SRBSDV P8 protein. CBB: Coomassie brilliant blue staining. **(D)** Representative images of mock-inoculated or *rice stripe virus* (RSV)-infected NIP and line 5 plants. Bar = 5 cm (top). Bar = 1 cm (bottom). **(E)** The percentages of RSV-infected NIP and line 5 plants. **(F)** Detection of RSV CP RNA expression levels by quantitative RT-PCR. *ρ < 0.05, **ρ < 0.01 by the Student’s *t* test.

To provide mechanistic insights into the effects of *OsV-ATPase d* on mediating ABA and JA biosynthesis in rice, the expression levels of key genes involved in the biosynthetic and signal transduction pathways of ABA and JA biosynthesis were retrieved from the transcriptomic dataset. As shown in **Figure 5**, the expression of the key genes *OsNCED4* (LOC_Os07g0154201) and *OsNCED5* (LOC_Os12g0617250) in ABA biosynthesis and of *OsSWEET15* (LOC_Os02g0513100) in the ABA signal transduction pathway (Teng et al., 2014; Yang et al., 2018a; Huang et al., 2019; ) were significantly upregulated in *OsV-ATPase d* knockout rice. The expression of *OsPAO7* (LOC_Os09g0368500), which is involved in ABA catabolism (Liu et al., 2014), was significantly downregulated in *OsV-ATPase d* knockout rice. **Figure 6** also shows that the expression of the key gene *OsAOS3* (LOC_Os02g0218700) in the JA biosynthetic pathway (Haga and Iino, 2004) was significantly upregulated in *Osv-ATPase d* knockout rice, and *OsJAZ5* (LOC_ Os07g0153000) and *OsCM-LOX1* (LOC_Os12g0559934) involved in JA signal transduction were the negative regulators of JA biosynthesis (Wang et al., 2008; Singh et al., 2015; ) and were significantly downregulated. Taken together, these results suggest that *OsV-ATPase d* is involved in the synergistic regulation of JA/ABA biosynthesis.

### Knockout of *OsV-ATPase d* mediates virus resistance in rice

Transcriptomic and plant hormone biosynthesis analysis showed that *OsV-ATPase d* may mediate resistance in rice. Three replicates of editing line 5 were evaluated for resistance against SRBSDV and RSV. SRBSDV disease symptom observations showed that at 30 dpi, the NIP plants showed more severe stunting upon SRBSDV infection (**Figure 6A**), and the SRBSDV disease incidence and accumulation of SRBSDV virions in the wild-type plants were significantly higher than those in the editing line 5 plants (**Figures 6B, C**). In contrast, the editing line 5 plants displayed higher susceptibility to RSV than the wild-type plants (**Figures 6D-F**). Further analyses showed that editing line 5 showed no significant effect on virus-transmitting vector infestation. These results indicated that *OsV-ATPase d* can differentially regulate rice resistance to SRBSDV and RSV infection.

## Discussion

In this study, we showed that knocking out a rice gene encoding V-ATPase d, subunit d of the membrane-embedded V0 complex of V-ATPase, differentially mediated resistance against the plant RNA viruses RBSDV and RSV and phytohormone biosynthesis in rice. To the best of our knowledge, this is the first report of a protein with a classical function as a proton pump for proton translocation in the regulation of vacuolar acidification being involved in synergistic regulation of JA/ABA biosynthesis and the mediation of plant defense against plant RNA viruses.

CRISPR/Cas9 is a novel tool for targeted mutagenesis that evolved from a type II bacterial immune system and has been well documented to edit crop genomes, including rice, with high efficiency (Jiang and Doudna, 2017; Liu et al., 2017; Hussain et al., 2018). The gene mutations, including nucleotide insertions, deletions and mutations, induced by CRISP/Cas9 in the rice genome were passed to the next generation (T1) following classic Mendelian laws, without any detectable novel mutations or reversions (Zhang et al., 2014; Ishizaki, 2016). Similar gene mutations, including nucleotide insertions, deletions and mutations, were induced in edited *OsV-ATPase d* by CRISP/Cas9 (**Figure 1B**), and the edited *OsV-ATPase d* could be passed to the next generation (T1) without novel mutations or reversions; however, whether the edited *OsV-ATPase d* is passed to the next generation (T1) following classic Mendelian laws was not verified in this study.

In eukaryotes, V-ATPase comprises V1 and V0 sections; V1 is located on the cytosolic side of the membrane and catalyses ATP hydrolysis, while V0 is a membrane-integral domain responsible for proton translocation, which consists of subunits a-d (Jefferies et al., 2008; Schumacher and Krebs, 2010; ). Loss of subunit d of V-ATPase in mung bean (*Vigna radiata*) impaired proton translocation across the tonoplast membrane, but it did not affect the growth and development of mung bean plants (Ouyang et al., 2008). The opposite was found in zebrafish (*Brachydanio rerio*), although normal melanocytes in early developmental stages later became pale and fragmented in *V-ATPase d* knockout mutants (Ramos-Balderas et al., 2013). Similar to mung bean, knocking out *OsV-ATPase d* had no detrimental impact on the growth and development of rice (**Figure 1** and **Table 1**), suggesting that *V-ATPase d* probably possesses multiple functions and differentially mediates development differently across eukaryotes.

The classical function of V-ATPases is mediating the pH of many intracellular organelles, including tonoplasts, vacuoles and endosomes, which is crucial for the fate of functional proteins from viruses. In plant−virus interactions, the extensive or conserved pathway of plant viruses inhibits the activity of V-ATPase, which results in an increase in the vacuolar pH and impairs the capacity of degradation proteins encoded by plant viruses in many intracellular organelles in plants. This reduced capacity promotes infection by many plant viruses, including BSMV, LRSV, CMV, and PVX (Yang et al., 2021). V-ATPase activity is also critical in mammal−virus interactions but is different from that of plant−virus interactions (Hunt et al., 2011; Jang et al., 2018). The V-ATPases of mammalian endosomes play pivotal roles in the successful entry and release of the viral genome into the cytoplasm for most human viruses, including HCoV-NL63, influenza viruses, ZIKV, DENV, and SINV (Hunt et al., 2011; Jang et al., 2018; Kao et al., 2018; Milewska et al., 2018). For the first time, this study provided a novel and third plant−virus interaction mechanism in which subunit d of V-ATPase in rice differentially mediates plant RNA viruses, which is reminiscent of previous document that plants with *Rice black-streaked dwarf virus* (RBSDV) infection were more resistant to subsequent challenge by SRBSDV, but more susceptible to RSV (Zhang et al., 2019). The distinct resistance against different plant RNA viruses probably through synergistic regulation of JA/ABA biosynthesis (**Table 2** and **Figure 6**). It is well documented that both JA and ABA are crucial for resistance against plant RNA viruses (Xie et al., 2018; Hu et al., 2020; Yang et al., 2020; ). Activating JA pathway would enhance the resistance to RSV in rice (Yang et al., 2020), however ABA negatively modulates plant defense against RBSDV infection by suppressing JA biosynthesis (Xie et al., 2018). Subunit d of V-ATPase in rice is also involved in mediating ABA, JA and auxin biosynthesis (Forgac, 2007). Previous studies have demonstrated that auxin-enhanced H^+^-pumping lowers the cell wall pH, activates pH-sensitive enzymes and proteins within the wall, and initiates cell-wall loosening and extension growth (Hager, 2003). Auxin can also decrease apoplastic pH regulated by V-ATPase to increase the expansion of conical cells in the flowers of angiosperm species (Dang et al., 2020). However, the interaction between ABA/JA and V-ATPase has received little attention and remains ambiguous. Therefore, uncovering the detailed molecular mechanisms of subunit d of V-ATPase in the rice-SRBSDV/RSV interaction with synergistic regulation of JA/ABA biosynthesis would provide novel insight into the function of V-ATPase in plant-RNA virus interactions.

## Conclusions

Altogether, the *OsV-ATPase d* knockout mutant of rice showed different levels of resistance to important viruses, SRBSDV and RSV, and did not show any detrimental effects on plant growth or yield productivity. This study indicates that *OsV-ATPase d* can be selected as a potential target for resistance breeding in rice. This study also paves the way for uncovering the novel molecular mechanisms of V-ATPase functioned in the rice-SRBSDV/RSV interaction and modulating plant hormones, which likely to dig out potential genes for viruses resistant breeding by CRISP/Cas9 system.

## Data availability statement

The datasets presented in this study can be found in online repositories. The names of the repository/repositories and accession number(s) can be found in the article/**Supplementary Material**.

## Author contributions

QL, XL, XY, methodology and investigation. TZ, YXL, YZ, and YLan, methodology and formal analysis. DZ and LZ, conceptualization and funding acquisition. LL, SZ, and YLiu, project administration, visualization, funding acquisition, writing – review and editing. All authors contributed to the article and approved the submitted version.
