# Supplementary material for: CRISPR/Cas9-mediated gene editing of vacuolar ATPase subunit d mediates phytohormone biosynthesis and virus resistance in rice
Source: Front Plant Sci. 2023 Feb 1;14:1122978. doi: 10.3389/fpls.2023.1122978 (PMC9929465; doi:10.3389/fpls.2023.1122978)
Supplement: Supplementary file 1 [file DataSheet_1.zip › Original data/Figure1 verification of editing v-ATPase d.docx]

**The sequences of V-ATPase d by Sangor sequencing**

>NIP （sgRNA1: 698-720 bp, marked as blue; sgRNA2: 755-774 bp, marked as red）

TTTAACCAATAGGCCGAAATCGGCAAAATCCCTTATAAATCAAAAGAATAGACCGAGATAGGGTTGAGTGTTGTTCCAGTTTGGAACAAGAGTCCACTATTAAAGAACGTGGACTCCAACGTCAAAGGGCGAAAAACCGTCTATCAGGGCGATGGCCCACTACGTGAACCATCACCCTAATCAAGTTTTTTGGGGTCGAGGTGCCGTAAAGCACTAAATCGGAACCCTAAAGGGAGCCCCCGATTTAGAGCTTGACGGGGAAAGCCGGCGAACGTGGCGAGAAAGGAAGGGAAGAAAGCGAAAGGAGCGGGCGCTAGGGCGCTGGCAAGTGTAGCGGTCACGCTGCGCGTAACCACCACACCCGCCGCGCTTAATGCGCCGCTACAGGGCGCGTCCATTCGCCATTCAGGCTGCGCAACTGTTGGGAAGGGCGATCGGTGCGGGCCTCTTCGCTATTACGCCAGCTGGCGAAAGGGGGATGTGCTGCAAGGCGATTAAGTTGGGTAACGCCAGGGTTTTCCCAGTCACGACGTTGTAAAACGACGGCCAGTGAATTGTAATACGACTCACTATAGGGCGAATTGGGCCCTCTAGATGCATGCTCGAGCGGCCGCCAGTGTGATGGATATCTGCAGAATTGCCCTATGTACGGGTGGGAGATGCTCTCGTTCAACATCCACGACGGCTTCCTGGAGGCCATCGTGCGGGGGAACCGGTCGGGCCTCCTCACCGCCGCCGACTACAACAACCTCTGCCAGTGCGAGAACCTCGACGACGTCAAGATGCACCTCACCGCCACCGAGTACGGCCCCTACCTCCAGAACAAGGGCAATTCCAGCACACTGGCGGCCGTTACTAGTGGATCCGAGCTCGGTACCAAGCTTGGCGTAATCATGGTCATAGCTGTTTCCTGTGTGAAATTGTTATCCGCTCACAATTCCACACAACATACGAG

>line 2 （sgRNA1: 698-719 bp, marked as blue; sgRNA2: 755-774 bp, marked as red）

TTTAACCAATAGGCCGAAATCGGCAAAATCCCTTATAAATCAAAAGAATAGACCGAGATAGGGTTGAGTGTTGTTCCAGTTTGGAACAAGAGTCCACTATTAAAGAACGTGGACTCCAACGTCAAAGGGCGAAAAACCGTCTATCAGGGCGATGGCCCACTACGTGAACCATCACCCTAATCAAGTTTTTTGGGGTCGAGGTGCCGTAAAGCACTAAATCGGAACCCTAAAGGGAGCCCCCGATTTAGAGCTTGACGGGGAAAGCCGGCGAACGTGGCGAGAAAGGAAGGGAAGAAAGCGAAAGGAGCGGGCGCTAGGGCGCTGGCAAGTGTAGCGGTCACGCTGCGCGTAACCACCACACCCGCCGCGCTTAATGCGCCGCTACAGGGCGCGTCCATTCGCCATTCAGGCTGCGCAACTGTTGGGAAGGGCGATCGGTGCGGGCCTCTTCGCTATTACGCCAGCTGGCGAAAGGGGGATGTGCTGCAAGGCGATTAAGTTGGGTAACGCCAGGGTTTTCCCAGTCACGACGTTGTAAAACGACGGCCAGTGAATTGTAATACGACTCACTATAGGGCGAATTGGGCCCTCTAGATGCATGCTCGAGCGGCCGCCAGTGTGATGGATATCTGCAGAATTGCCCTATGTACGGGTGGGAGATGCTCTCGTTCAACATCCACGACGGCTTCCTGGAGGCCATCGTGAGGGGAACCGGTCGGGCCTCCTCACCGCCGCCGACTACAACAACCTCTGCCAGTGCGTGAACCTCGGCGACGTCAAGATGCACCTCACCGCCACCGAGTACGGCCCCTACCTCCAGAACAAGGGCAATTCCAGCACACTGGCGGCCGTTACTAGTGGATCCGAGCTCGGTACCAAGCTTGGCGTAATCATGGTCATAGCTGTTTCCTGTGTGAAATTGTTATCCGCTCACAATTCCACACAACATACGAG

>line 5 （sgRNA1: 698-721 bp, marked as blue; sgRNA2: 755-773 bp, marked as red）

TTTAACCAATAGGCCGAAATCGGCAAAATCCCTTATAAATCAAAAGAATAGACCGAGATAGGGTTGAGTGTTGTTCCAGTTTGGAACAAGAGTCCACTATTAAAGAACGTGGACTCCAACGTCAAAGGGCGAAAAACCGTCTATCAGGGCGATGGCCCACTACGTGAACCATCACCCTAATCAAGTTTTTTGGGGTCGAGGTGCCGTAAAGCACTAAATCGGAACCCTAAAGGGAGCCCCCGATTTAGAGCTTGACGGGGAAAGCCGGCGAACGTGGCGAGAAAGGAAGGGAAGAAAGCGAAAGGAGCGGGCGCTAGGGCGCTGGCAAGTGTAGCGGTCACGCTGCGCGTAACCACCACACCCGCCGCGCTTAATGCGCCGCTACAGGGCGCGTCCATTCGCCATTCAGGCTGCGCAACTGTTGGGAAGGGCGATCGGTGCGGGCCTCTTCGCTATTACGCCAGCTGGCGAAAGGGGGATGTGCTGCAAGGCGATTAAGTTGGGTAACGCCAGGGTTTTCCCAGTCACGACGTTGTAAAACGACGGCCAGTGAATTGTAATACGACTCACTATAGGGCGAATTGGGCCCTCTAGATGCATGCTCGAGCGGCCGCCAGTGTGATGGATATCTGCAGAATTGCCCTATGTACGGGTGGGAGATGCTCTCGTTCAACATCCACGACGGCTTCCTGGAGGCCCATCGTGCGGGGGAACCGGTCGGGCCTCCTCACCGCCGCCGACTACAACAACCTCTGCCAGTGCGAGAACCTCGACACGTCAAGATGCACCTCACCGCCACCGAGTACGGCCCCTACCTCCAGAACAAGGGCAATTCCAGCACACTGGCGGCCGTTACTAGTGGATCCGAGCTCGGTACCAAGCTTGGCGTAATCATGGTCATAGCTGTTTCCTGTGTGAAATTGTTATCCGCTCACAATTCCACACAACATACGAG
