# Supplementary figures and images for: CRISPR/Cas9-mediated gene editing of vacuolar ATPase subunit d mediates phytohormone biosynthesis and virus resistance in rice
Source: Front Plant Sci. 2023 Feb 1;14:1122978. doi: 10.3389/fpls.2023.1122978 (PMC9929465; doi:10.3389/fpls.2023.1122978)

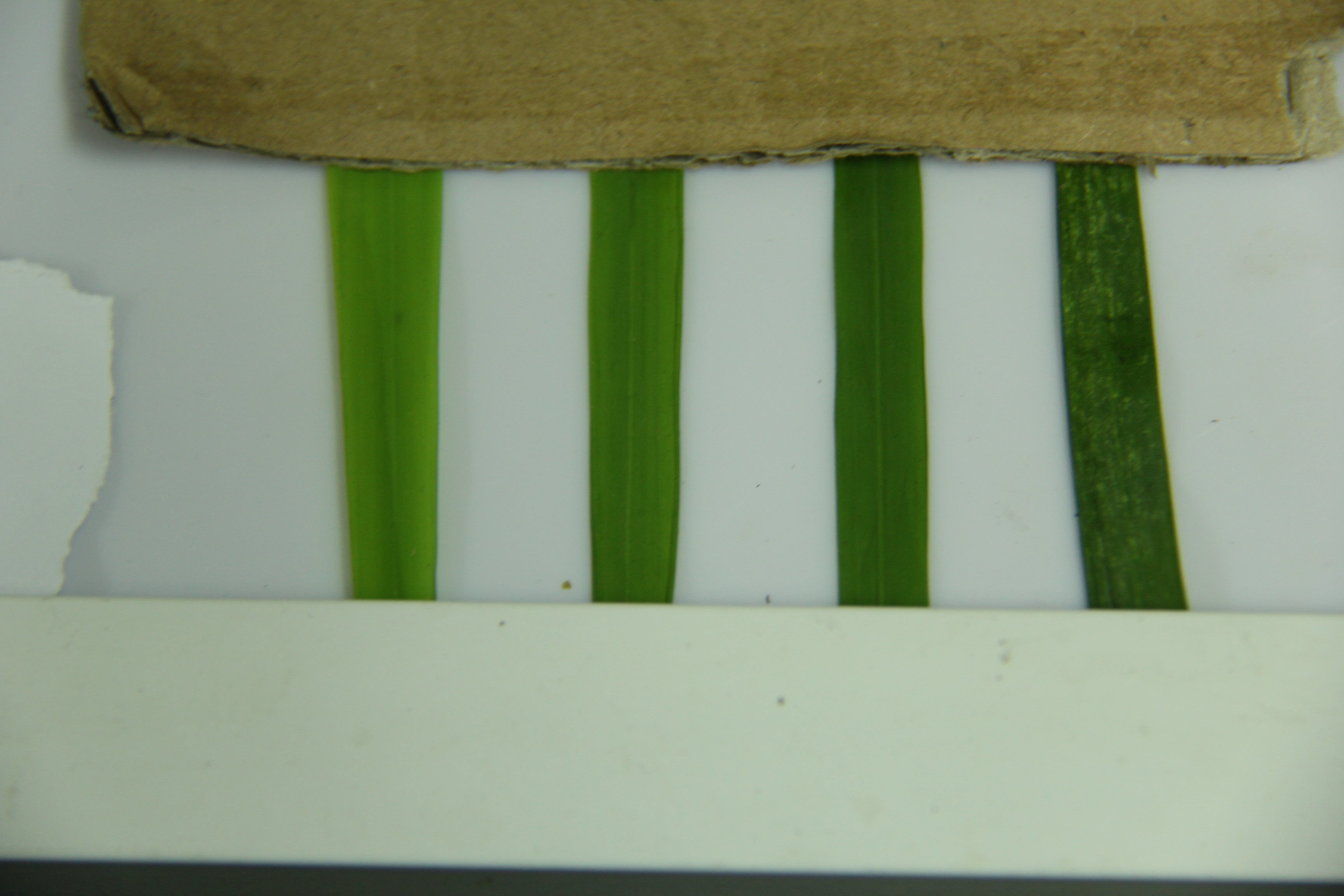

Supplement: Supplementary file 1 [file DataSheet_1.zip › Original data/Figure 6/Figure 6a-down 30dpi leaves symptom of edited line5.JPG]

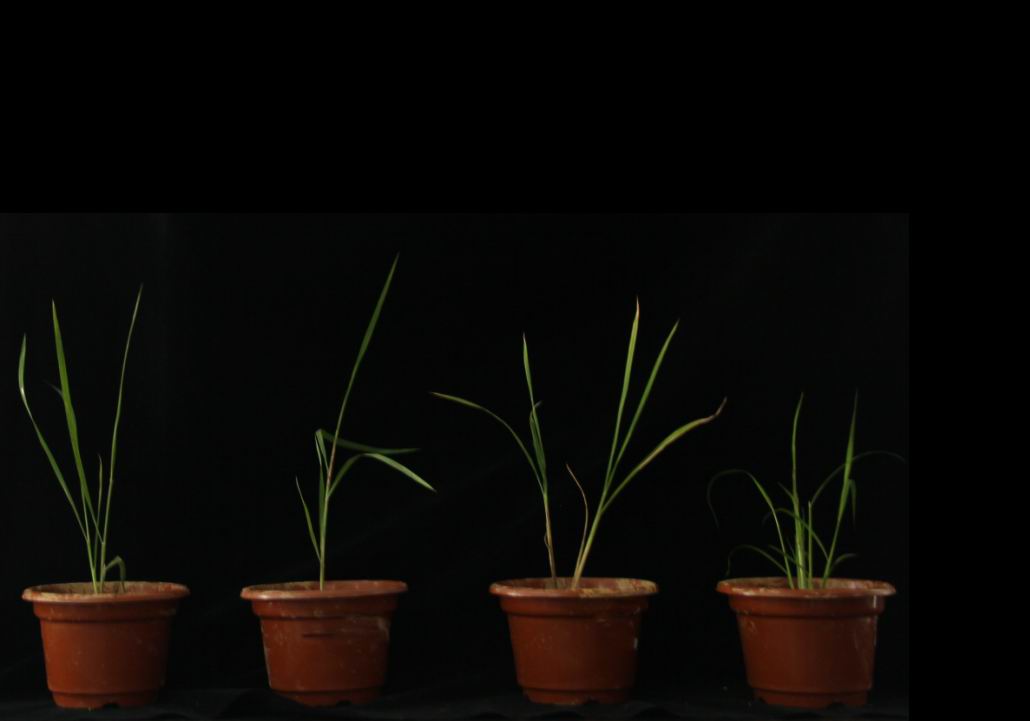

Supplement: Supplementary file 1 [file DataSheet_1.zip › Original data/Figure 6/Figure 6a-up 30dpi edited line5.jpg]

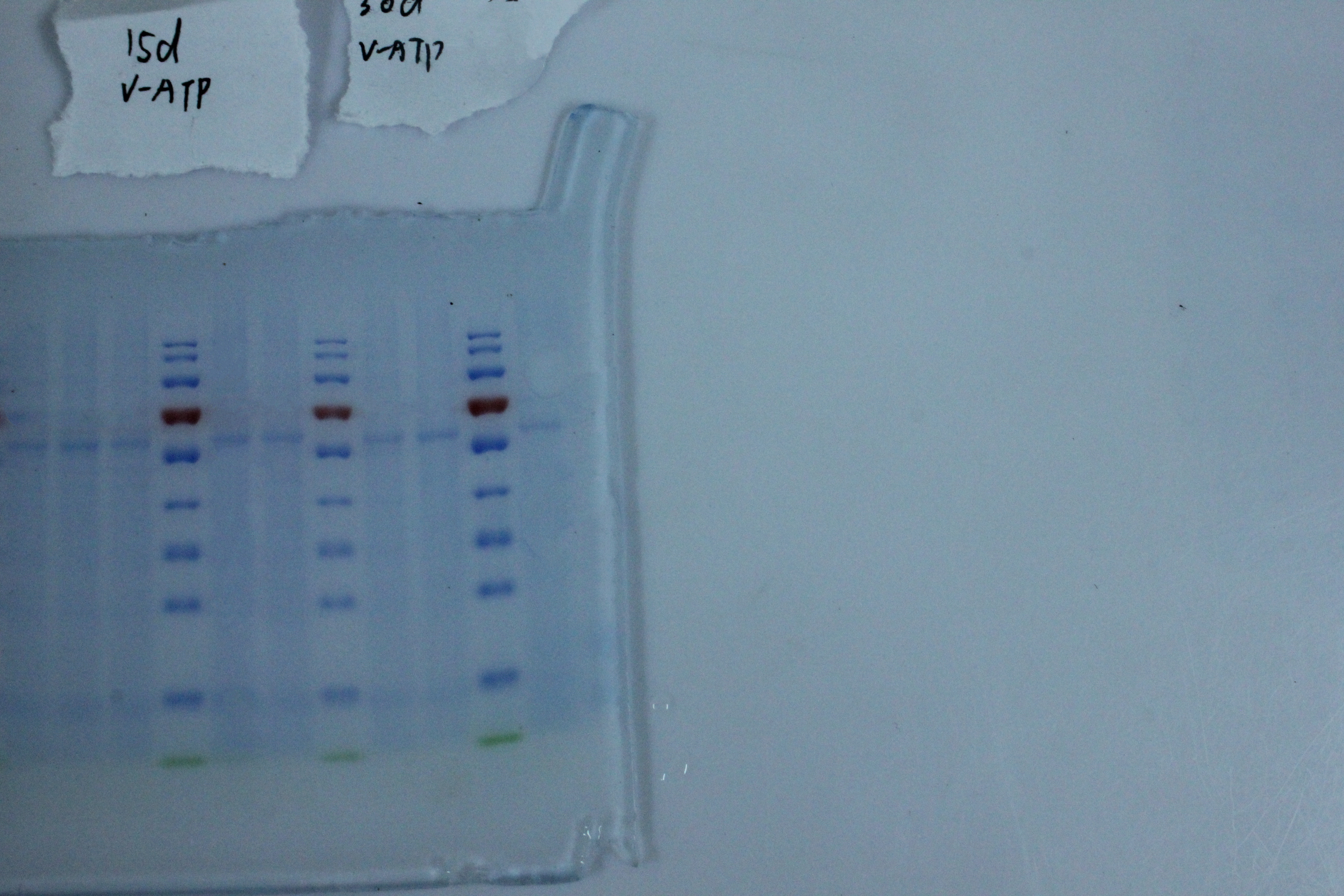

Supplement: Supplementary file 1 [file DataSheet_1.zip › Original data/Figure 6/Figure 6c-down SDS-PAGE for western blotting of SRBSDV.JPG]

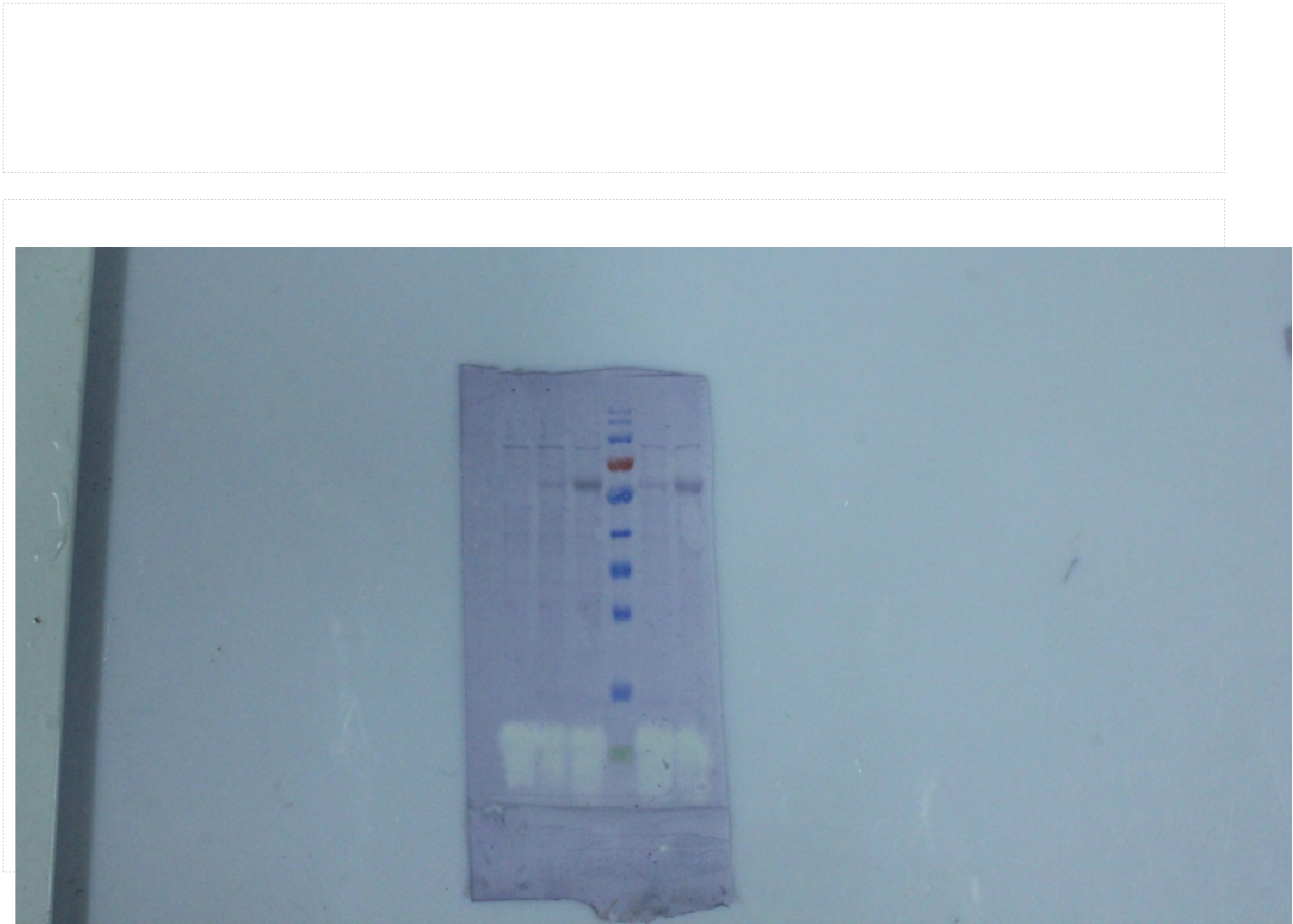

Supplement: Supplementary file 1 [file DataSheet_1.zip › Original data/Figure 6/Figure 6c-down Western blotting of SRBSDV.png]

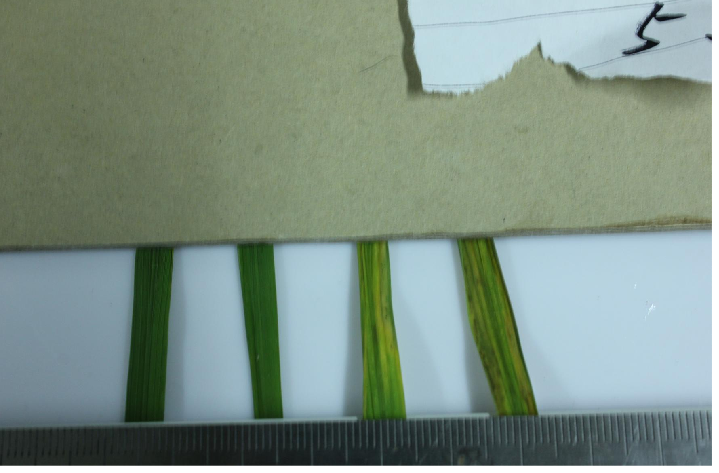

Supplement: Supplementary file 1 [file DataSheet_1.zip › Original data/Figure 6/Figure 6d-down.png]

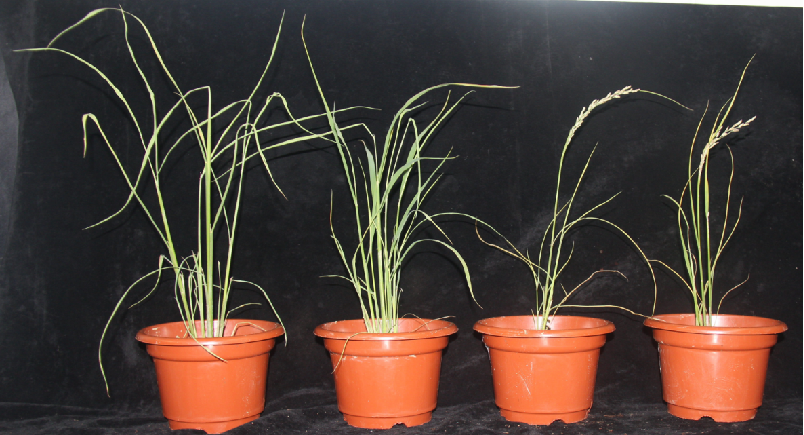

Supplement: Supplementary file 1 [file DataSheet_1.zip › Original data/Figure 6/Figure 6d-up.jpg]

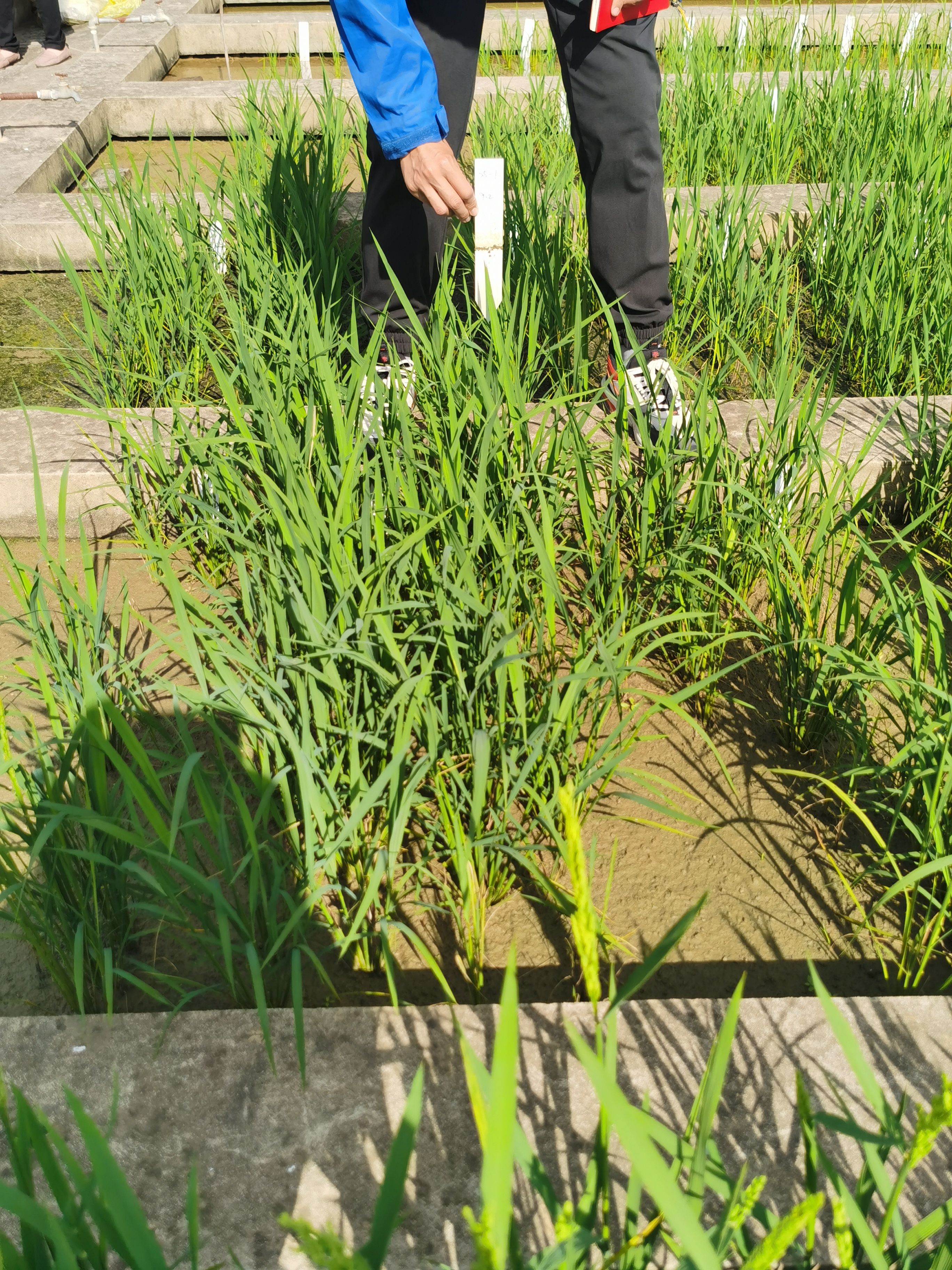

Supplement: Supplementary file 1 [file DataSheet_1.zip › Original data/Figure 6/Figure 6e.jpg]
