# Supplementary material for: CRISPR/Cas9-mediated gene editing of vacuolar ATPase subunit d mediates phytohormone biosynthesis and virus resistance in rice
Source: Front Plant Sci. 2023 Feb 1;14:1122978. doi: 10.3389/fpls.2023.1122978 (PMC9929465; doi:10.3389/fpls.2023.1122978)
Supplement: Supplementary file 2 [file DataSheet_2.doc]

**Supplementary information**

**Additional file 1: Table S1 Primers used in this study**

**Additional file 2: Table S2 The data of transcriptome in *Osv-ATPase d* knocking-out line 5 and NIP rice**

**Additional file 3: Figure S1 Gene Ontology (GO) analysis of the upregulated differentially expressed genes**

**Additional file 4: Figure S2 Gene Ontology (GO) analysis of the downregulated differentially expressed genes**

**Additional file 1. Table S1 Primers used in this study**

**Table S1 Primers used in this study**

| Primer name | Sequence |
| --- | --- |
| **For vector construction** | |
| Crispr/Osv-ATPase d/1F | AGATGATCCGTGGCAGACCCAGTCCTTCCCATCGTGCGGGGGAACCGGTCGG |
| Crispr/Osv-ATPase d/1R | GCATAGCTCTAAAACTTGGGGAAGGACCCGACCGGTTCCCCCGCACGATG |
| Crispr/Osv-ATPase d/2F | AGATGATCCGTGGCAGACCCAGTCCTTCCCCCAGTGCGAGAACCTCGACGACG |
| Crispr/Osv-ATPase d/2R | GCATAGCTCTAAAACTTGGGGAAGGACTGGGCGTCGTCGAGGTTCTCGCACTGG |
| **For verification of Crispr mutants** | |
| Osv-ATPase d/F | ATGTACGGGTGGGAGATGCT |
| Osv-ATPase d/R | GTTCTGGAGGTAGGGGCC |
| **For cas9 detection** | |
| 35S-F1 | CAAGACCCTTCCTCTATATAAGGA |
| zCas9-951R | GAGGTTATCCAGGTCATCG |
| **For RT-qPCR** | |
| qUbiquitin/F | GCTCCGTGGCGGTATCAT |
| qUbiquitin/R | CGGCAGTTGACAGCCCTAG |
| SRBSDV/qCP/F | CGCGTCATCTCAAAACTACAG |
| SRBSDV/qCP/R | TTTGTCAGCATCTAAAGCGC |
| RSV/qCP/F | AGTGCTGATCGTATTGACAGA |
| RSV/qCP/R | GATGAAGTACACAACTGGTC |
| qOsLBD12/F | G GTAATCGTTG ATGGCCAAAT C |
| qOsLBD12/R | GATTGATTTTCATAGACGGTGC |
| qOsATL79/F | GTTGGGACAG CGATGATCAG GT |
| qOsATL79/R | TAAGGATTACTCCATTGTCCTG |
| qOsGSTT3/F | GGCAGCCATGGAGCACAAGGAG |
| qOsGSTT3/R | ATACTCTTCAATGCCATCAC |
| qOsMYB5/F | AGCTCAACCAGCGCTTCTAC |
| qOsMYB5/R | CCTTCTTGAGCGACTCCATC |
| qOsAOX1B/F | GAATCGGCGAACTTTACAGC CG |
| qOsAOX1B/R | TGGGCCCGGAGATCACTGTTCCG |
| qOsRGA5/F | GGAAGATCGA AATCGATGTG TCCG |
| qOsRGA5/R | ATATTAGGATTCTGACCGACCGG |
| qOsNCED4/F | ACATCCGAGCTCCTCGTCGTGAA |
| qOsNCED4/R | TTGGAAGGTGTTTTGGAATGAACCA |
| qOsCHX15/F | CCCTTCCACAAGTCGTCGGACG |
| qOsCHX15/R | AAGTACAACGCCACCCGCTGCA |
| qOsCKX1/F | GG TTGGCCGGCG ACGTCTTTGA |
| qOsCKX1/R | CCTTGGAGCACGTCACCGTCTC |
| qOsWRKY27/F | TCCGAAGCAA CAGGAGGTGC TT |
| qOsWRKY27/R | CGGTCATCAGCCAGCGAGCGAA |

**Additional file 2: Tables S2 The data of transcriptome in *Osv-ATPase d* knocking-out line 5 and NIP rice**

Table S2 The data of transcriptome in *Osv-ATPase d* knocking-out line 5 and NIP rice

| Sample | Raw Reads | Raw Bases | Clean Reads | Clean Bases | Valid Bases | GC |
| --- | --- | --- | --- | --- | --- | --- |
| NIP_1 | 47.32M | 7.10G | 46.31M | 6.53G | 91.93% | 52.83% |
| NIP_2 | 45.18M | 6.78G | 44.28M | 6.26G | 92.33% | 52.54% |
| NIP_3 | 47.12M | 7.07G | 46.08M | 6.48G | 91.73% | 52.78% |
| line 5_1 | 48.50M | 7.28G | 47.46M | 6.71G | 92.18% | 52.71% |
| line 5_2 | 50.21M | 7.53G | 49.14M | 6.91G | 91.73% | 52.21% |
| line 5_3 | 44.94M | 6.74G | 43.98M | 6.23G | 92.48% | 52.45% |

**Additional file 3: Figure S1 Gene Ontology (GO) analysis of the upregulated differentially expressed genes**


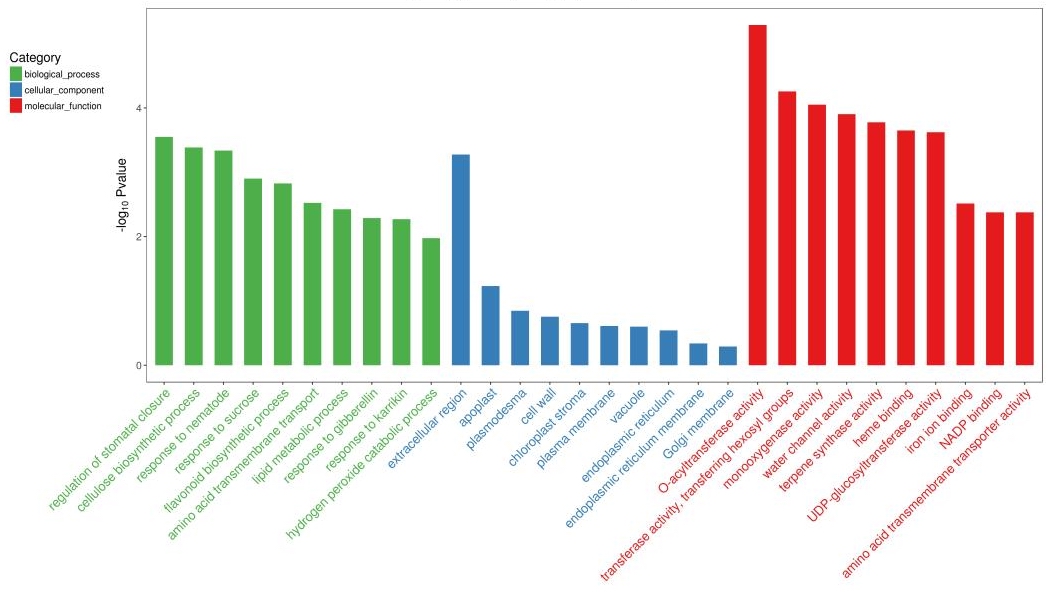


**Figure S1 Gene Ontology (GO) analysis of the upregulated differentially expressed genes**

**Additional file 4: Figure S2 Gene Ontology (GO) analysis of the downregulated differentially expressed genes**


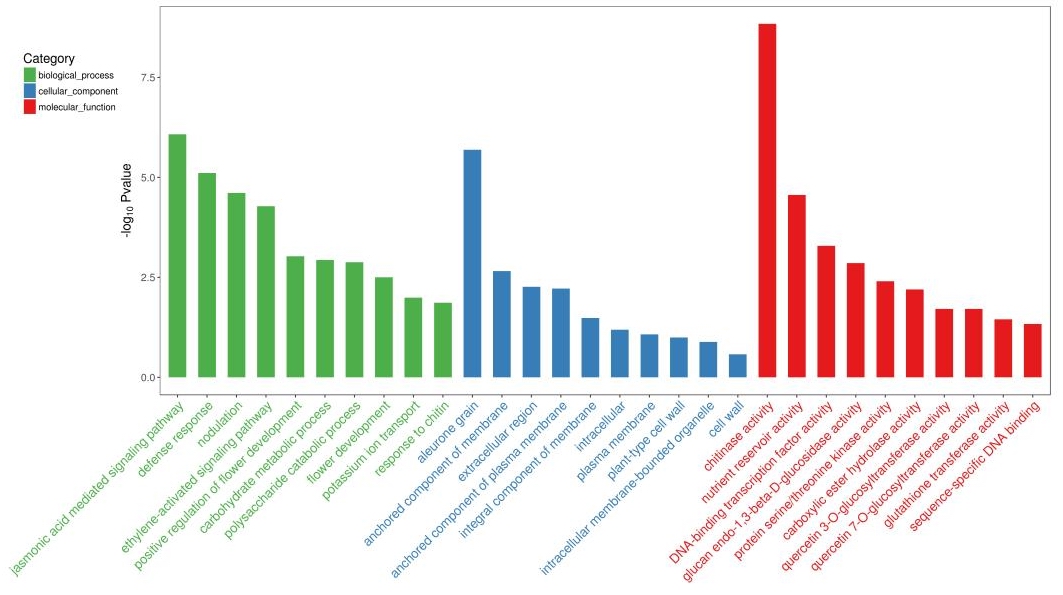


**Figure S2 Gene Ontology (GO) analysis of the downregulated differentially expressed genes**
